# T-cell receptor chain Vbeta subunit staining to quantify the malignant clone in adult T-cell leukemia

**DOI:** 10.1186/1742-4690-12-S1-P45

**Published:** 2015-08-28

**Authors:** Aileen G Rowan, Paul Fields, Graham P Taylor, Charles RM Bangham

**Affiliations:** 1 Section of Virology, Department of Medicine, Imperial College London, UK; 2 Guy's and St Thomas’ Hospital, London, UK

## 

Adult T cell leukemia (ATL) is typically characterised by expansion of a single dominant malignantly transformed HTLV-1-infected clone. Clinically, the abundance of this clone can be estimated by flow cytometric assessment of CD4, CD25 and CD7 expression, and, more recently, by quantitative LM-PCR analysis of viral integration site. In addition to the unique proviral integration site, malignant clones arising from a single infection event also share an identical T cell receptor (TCR) sequence, utilising one of a potential 47 TCR Vbeta subunit genes. Using multiparametric flow cytometry, we quantified the frequency of peripheral T cells bearing each of 24 TCR Vbeta subunits in a cohort of individuals with adult T cell leukemia or lymphoma (n=26) at various stages of disease progression/remission. To establish an appropriate baseline, we also analysed T cells from asymptomatic carriers (n=11) and donors with HAM/TSP (n=13). Vbeta-specific antibodies stained 75% of CD3+ cells in donors without malignancy, and the median size of the largest single subset (CD4+Vbeta2+) was 7.6 % of CD3+ cells. We detected putative T cell clones which exceeded 20% of CD3+ T cells only in individuals with active chronic or acute leukemia (n=17), reaching frequencies of up to 96% of CD3+ T cells. With this method, we quantified the expression on clonally expanded populations of the putative ATL cell markers CD25, CCR4, TSLC1, CD7 and CD127. Almost 1% of expanded clonal populations in ATL patients were CCR4+TSLC1+CD7–. Surprisingly, in most donors only 60% of the dominant clonal population expressed high levels of CD25. This flow cytometric protocol is a potentially powerful method to both monitor disease progression and response to therapy, providing fast and accurate quantification of clonally expanded populations. We are currently employing this technique to assay lysis of expanded clones by autologous cytotoxic T lymphocytes in patients with ATL.

